# Establishing percentiles for blood pressure based on absolute height for children and adolescents

**DOI:** 10.1186/s12887-020-02489-9

**Published:** 2021-01-08

**Authors:** Marco Cossio-Bolaños, Rubén Vidal-Espinoza, Felipe Castelli Correia de Campos, José Sulla-Torres, Wilbert Cossio-Bolaños, Cynthia Lee Andruske, Camilo Urra Albornoz, Rossana Gómez Campos

**Affiliations:** 1 Universidad Católica del Maule, Av San Miguel 3605, Talca, Chile; 2 Universidad Católica Silva Henríquez, Santiago, Chile; 3 Universidad del Bío Bío, Chillán, Chile; 4 Universidad Nacional de San Agustín de Arequipa, Arequipa, Perú; 5 Universidad Privada San Juan Bautista, Lima, Perú; 6 Centro de Investigación CINEMAROS, Arequipa, Perú; 7 Escuela de Kinesiología, Facultad de Salud, Universidad Santo Tomás, Talca, Chile

**Keywords:** Blood pressure, Percentiles, Height, Children, Adolescentes

## Abstract

**Background:**

Evaluating blood pressure (BP) is one element for diagnosing and preventing disease in student populations. The objectives of this research were to (a) identify the range of height for measuring BP adjusted for student populations and (b) propose percentiles for evaluating BP based on height.

**Methods:**

A cross-sectional study was carried out with 3,013 students. Weight, height, and diastolic (DBP) and systolic (SBP) blood pressure were evaluated. Body Mass Index (BMI) was calculated. Height ranges of 5 and 10 cm were generated.

**Results:**

R^2^ values for height ranges of 5 cm consisted of [normotensive: DBP (R^2^ = 10 to 13%) and SBP (R^2^ = 14 to 20%), and for hypertensive: DBP (R^2^ = 0.07 to 15%) and for SBP (R^2^ = 29 to 32%)]. For height ranges of 10 cm, values included: [normotensive: DBP (R^2^ = 10 to 15%), and SBP (R^2^ = 15 to 21%) and for hypertensive: DBP (R^2^ = 0.07 to 16%) and SBP (R^2^ = 29 to 35%)]. For 5 cm height ranges, diferences occurred between both sexes for DBP (in 5 height ranges from 123 to 148 cm and 158 to 168 cm) and for the SBP (in 6 height ranges from 128 to 148 cm and from 158 to 168 cm). In the 10 cm categories, diferences appeared in DBP (from 138 to 148 cm) and in the SBP (from 158 to 168 cm).

**Conclusions:**

Height is a determinant for evaluating blood pressure, and height ranges of 10 cm are more suitable for children and adolescents. The proposed percentiles based on height ranges allowed assessment of the DBP and SBP suggest their use in epidemiological and educational contexts.

## Background

Evaluation of blood pressure (BP) and the prevention of hypertension in children and adolescents has become a priority worldwide [1]. Thus, until a few years ago, their inclusion in clinical practice during physical examinations was unusual [2]. Actually, it is widely known that evaluating BP in children and adolescent populations was an important component, not only for the pediatric examination, but also for prevention in medical examinations [3].

Identifying children with high blood pressure is difficult to establish. This is especially the case since determining factors exist, such as age, sex, ethnicity, specific racial height groups [4, 5], socioeconomic conditions, and lifestyle [6], among other factors.

In this sense, a number of simple and easy to use tools have emerged in clinical practice for detecting high BP in children and adolescents [7]. Generally, these are based fundamentally on percentile tables [8].

In fact, in their studies, some researchers have proposed references for diverse regions of the world. Some are based on the function of chronological age [1, 9] and others by height [2, 3, 10]. Many of these are cumbersome and impractical for daily use. In addition, some even incorporate a variable other than age in the presentation of their standards [11]. This makes it difficult to quickly detect high BP in children and adolescents.

Therefore, in the presence of a variety of methods that allow identification of elevated levels of BP in children and adolescents, recent studies have demonstrated that the measurement of absolute height divided into height ranges of 5 cm [12] and 10 cm [13] are practical tools and useful in detecting high BP in children and adolescents. Thus, the methods based on chronological age and proposed formulas are better suited for tall children [8].

As a result, based on these premises, this study was guided by the following objectives: (a) identify height ranges (5 and 10 cm) for detecting BP in children and adolescents better suited to students in the Maule Region and (b) propose percentiles for assessing BP based on age, sex, and absolute height. This information may be useful for researchers and health science professionals for developing specific strategies for the Maule Region.

## Methods

### Type of study and sample

A descriptive cross-sectional study was carried out with a sample of 3,013 students Chileans (Latinos – Latin Americans) between the ages of 5.0 and 18.9 years old. The simple population consisted of students from elementary and secondary municipal schools located in the Maule Region of Chile. The number of students in the study totaled 29,500 (17,410 males and 12,100 females). Probability proportional to size (PPS) sampling was used to select the sample. Stratified sampling proportional to the total number of students based on sex and age from the different schools was used to determine the final sample. Specifically, 12 schools were included, resulting in 10,2% [1832 (6.2%) males and 1,181 (4.0%) females] with a CI of 95%.

The study was conducted according to the Declaration of Helsinki for Human Subjects. In addition, the research was approved by the Ethics Committee from the Universidad Autónoma de Chile (certificate number 2413). Parents or guardians approved the evaluation of their children by signing informed consent. The students themselves also provided written consent to participate.

Students of both sexes from municipal schools included in this study ranged in age from 5.0 to 18.9 years old. Students excluded from the research were those taking medication or with any type of illness and/or symptom of one during the previous month and those not completing the anthropometric examinations (weight and/or height).

### Procedures

Data collection took place from March to August 2017. All evaluations were carried out in specified locations at each school (Department of Physical Education) during classes held from 8:00 a.m. to 12:00 noon, Monday to Friday. Prior to collecting information, each school was asked for permission to carry out the research. Based on the address, each school provided data, such as birth date, age, sex, and parent or guardian’s name. Anthropometric evaluations and BP were collected by 2 experienced health professionals.

The anthropometric evaluations were carried out with the students barefoot, wearing shorts and a shirt as suggested and described by Ross and Marfell-Jones [14]. Body weight was measured with a digital scale (Tanita, United Kingdom, Ltd.) with a scale of 0-150 kg and an accuracy of 100 g. Standing height was measured using a portable stadiometer (Seca & Co. KG, Hamburg, Germany) with a precision of 0.1 mm and a scale of 0–2.50 m. After every 10 subjects, the scale was reset, and the stadiometer was recalibrated. Body Mass Index (BMI = weight/standing height) was calculated.

BP (diastolic DBP and systolic SBP) was recorded based on the recommendations of the American Academy of Pediatrics (AAP) [15]. Each subject sat on a chair with his or her back against the back of a chair with feet planted on the floor and the right arm (unclothed) extended on a table (at the height of the heart). BP was taken twice at 1 min intervals between measurements. A certified mercury sphygmomanometer (Omron M6) [16] was used to measure the BP.

The cut-off points for BP were adopted according to those proposed by the United States Department of Health and Human Services [17]: normotensive < p90; pre-hypertensive ≥ p90 to p95; and hypertensive ≥ p95. To evaluate BP, height intervals based on age ranges were created and used according to those suggested by Banker et al. [12] and described by Chiolero et al. [5] in ranges of 5 cm and in ranges of 10 cm. In addition, 108 cm was established as the minimum height with 188 cm as the maximum height for males, and for females, 98 cm was the minimum and 178 cm the maximum height.

### Statistical analysis

Normalization of the data was carried out by using Kolmoronov Smirnov’s method. Descriptive statistical mean (X), standard deviation (SD), and ranges were used. Comparison of data between both sexes was carried out with a student t-test for independent samples. Pearson’s correlations were used to analyze the relationship between DBP and SBP with height to categorize normotensive and hypertensive BP for both sexes and for height ranges of 5 cm and 10 cm. In addition, the % of explication of R² was calculated. For all cases, *p* < 0.05 was adopted. These calculations were performed using SPSS 18.0. The LMS method was used based on three smoothed curves [L(t) Box-Cox transformation, M(t) median, and S(t) Coefficient of Variation] to create the percentiles [18]. For each sex, percentiles P50, P90, P95, and P97 were calculated for DBP and SBP for absolute height. LMS Chart Maker version 2.3 [19] was used to generate the curves.

## Results

The anthropometric variables and BP reflected in the sample of children and adolescents from the Maule Region are illustrated in Table 1. No significant differences in weight and height occurred from age 5 to 14 years old. However, from 15 to 18 years old, males were heavier and taller than the females (*p* < 0.05). For BMI, the significant differences were found in both sexes (*p* > 0.05). For DBP, males presented higher values from age 7 to 12 years old (*p* < 0.05). However, for the remaining ages, the values were relatively similar for both sexes (*p* > 0.059). For SBP, males showed values significantly higher than the females at ages 8 and 9 years old, and 11 and 12 years old, and from 15 to 18 years old (*p* < 0.05).

**Table 1:** Anthropometric characteristics of the children and adolescents studied

Age (years)		Weight (kg)		Height (cm)		BMI (kg/m2)		DBP (mmHg)		SBP (mmHg)	
	n	X	SD	X	SD	X	SD	X	SD	X	SD
Males											
5.0-5.9	64	21.5	3.3	113.5	6.0	16.6	1.8	61.8	13.8	97.9	14.1
6.0-6.9	77	26.0	6.6	120.1	6.1	17.9	3.8	61.7	13.7	97.6	12.5
7.0-7.9	63	31.0	7.0	127.3	6.6	19.0	3.1	64.8*	11.7	101.7	14
8.0-8.9	77	32.2	6.5	130.4	5.3	18.8	2.9	63.8*	9.5	103.0*	13
9.0-9.9	96	36.8	8.8	136	8.3	19.8	3.8	66.9*	11.4	106.4*	14.5
10.0-10.9	92	42.1	9.8	142.4	7.1	20.6	3.5	67.3*	10.6	106.2	12.8
11.0-11.9	89	47.2	10.8	147.9	7.7	21.5	4.0	69.8*	13.3	109.2*	15.7
12.0-12.9	106	50.4	10.9	154.3	8.2	20.7	4.0	69.9*	11.2	110.7*	16.7
13.0-13.9	157	54.7	10.2	160.8	8.5	20.7	2.9	68.1	12.1	110.8	14
14.0-14.9	180	60.0	11.0	166.1	6.6	21.7	3.7	68.3	11.7	112	15
15.0-15.9	182	64.5*	9.2	169.9*	7.0	22.3	3.0	69.2	10.1	114.3*	14.8
16.0-16.9	214	70.4*	12.8	171.5*	7.1	23.9	4.1	70.9	10.5	117.4*	15.8
17.0-17.9	279	71.7*	13.3	171.4*	6.4	24.4	4.1	73.5	12.7	121.8*	19.3
18.0-18.9	156	71.6*	11.0	172.0*	6.9	24.2	3.6	71.2	12.3	119.7*	20.5
Total	1832	56.1	19.2	157.4	19.5	21.9	4.2	69.1	12.1	112.5	17.5
Females											
5.0-5.9	55	22.1	3.5	113.7	5.2	17.0	2.2	62.2	12.0	97.5	12.6
6.0-6.9	58	25.7	5.4	119.4	5.8	17.9	2.9	60.1	10.7	98.6	10.4
7.0-7.9	52	29.1	6.0	126.2	6.2	18.1	2.5	61.4	9.5	101.1	12.4
8.0-8.9	52	32.9	7.5	130.2	6.6	19.2	3.4	61.7	8.1	99.2	11.1
9.0-9.9	76	36.5	8.0	137.4	6.8	19.2	3.1	63.9	8.8	101.6	10.9
10.0-10.9	116	42.1	9.1	144.0	8.0	20.2	3.2	65.2	8.7	105.4	12.3
11.0-11.9	81	47.2	9.0	150.9	7.3	20.7	3.6	65.6	9.4	104.4	12.9
12.0-12.9	110	54.0	10.7	156.0	6.2	22.1	4.0	66.1	12.0	108	12.7
13.0-13.9	75	56.3	10.3	158.6	7.3	22.4	3.6	69.0	10.3	110.9	12.0
14.0-14.9	93	59.3	11.6	158.6	6.9	23.5	3.8	69.5	11.1	112.0	16.1
15.0-15.9	81	60.4	10.5	159.6	4.2	23.7	3.9	69.0	9.1	110.3	13.2
16.0-16.9	114	62.8	12.1	159.2	5.5	24.7	4.6	71.5	9.6	113.1	13.3
17.0-17.9	141	64.1	13.9	158.5	4.9	25.4	4.9	72.3	12.4	111.1	16.4
18.0-18.9	77	59.7	9.6	157.8	5.7	24	3.6	72.4	10.7	109.7	14.1
Total	1181	50.5	17	149	15.6	22	4.6	67.4	11.1	107.3	14.2

Legend: * significant difference in relation to women, *X* Average, *SD* Standard deviation, *BMI* Body Mass Index, *SBP* Systolic blood pressure, *DBP* Diastolic blood pressure

The relationships between the DBP and SBP with height for each category classified for BP are shown in Table 2. The values for R and R^2^ were relatively similar when aligned by BP category for 5 cm and 10 cm.

**Table 2:** Relationship of height with BP by category in normotensive and hypertensive in children of both sexes

Categories	Males				Females			
	DBP (mmHg)		SBP (mmHg)		DBP (mmHg)		SBP (mmHg)	
	R	R^2^	R	R^2^	R	R^2^	R	R^2^
*5 cm categories*								
Normotensive	0.31	0.1	0.45	0.2	0.37	0.13	0.38	0.14
Hypertensive	0.27	0.07	0.57	0.32	0.39	0.15	0.53	0.29
*10 cm categories*								
Normotensive	0.32	0.1	0.46	0.21	0.39	0.15	0.39	0.15
Hypertensive	0.26	0.07	0.59	0.35	0.4	0.16	0.53	0.29

Legend: *SBP* Systolic blood pressure, *DBP* Diastolic blood pressure

**Fig. 1:**
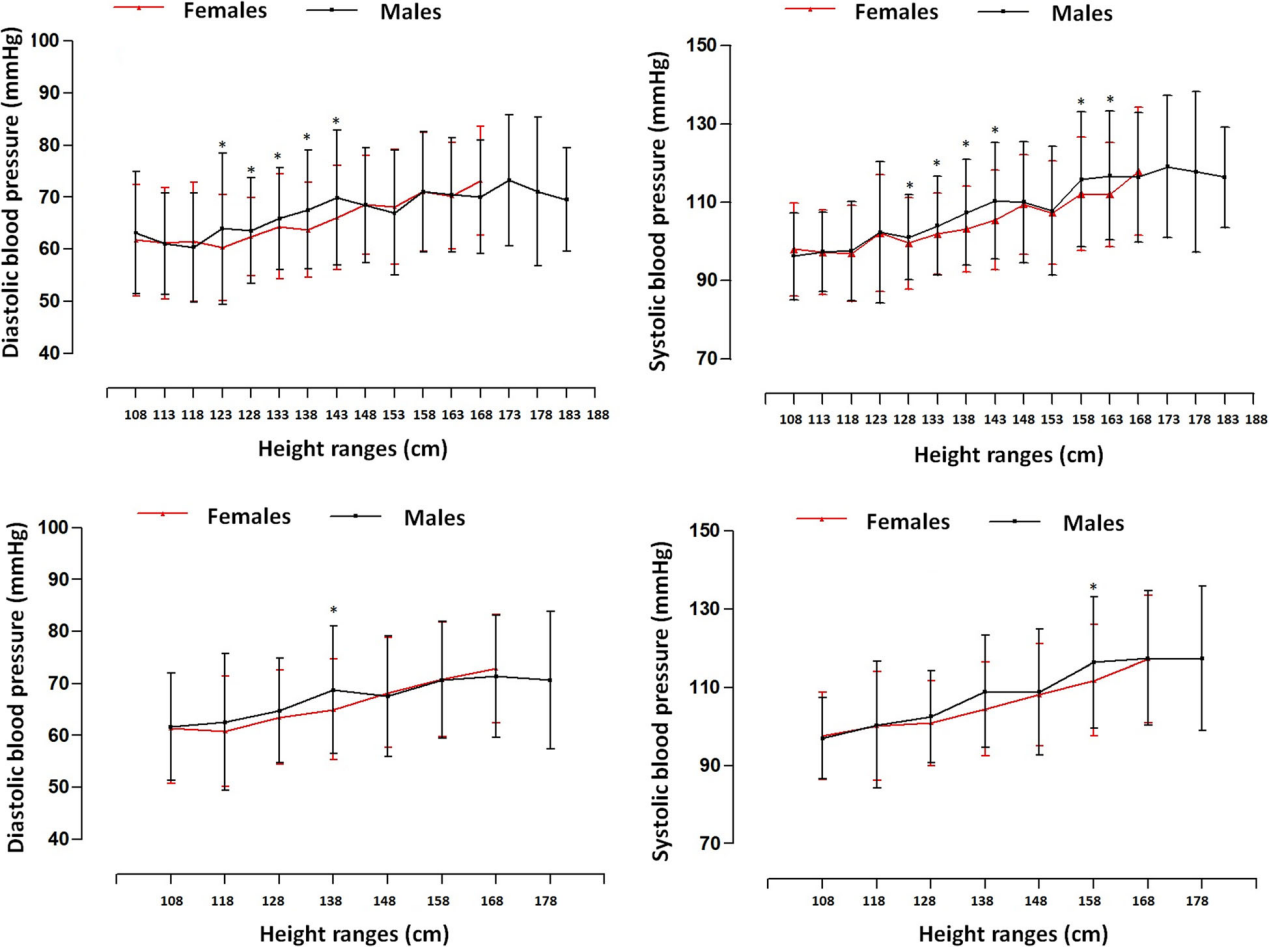
Comparison of the mean and ± SD values for Diastolic blood pressure and Systolic blood pressure by age ranges for both sexes (above 5 cm and below 10 cm)

Comparisons for BP adjusted for absolute height for ranges of 5 and 10 cm are depicted in Fig. 1. Significant differences occurred between both sexes when SBP and DBP were aligned by ranges of 5 cm: ranges 128 to 133 cm; from 133 to 138 cm; from 138 to 143 cm; and from 143 to 148 cm (*p* < 0.05). For the SBP, differences continued in height ranges: 158 to 163 and 168 to 173 cm (*p* < 0.05). Furthermore, differences were observed in DBP in the range of 123 to 128 cm (*p* < 0.05). For the 10 cm categories, differences emerged in DBP in the range of 138 to 148 cm (*p* < 0.05) while in the SBP, the range was 158 to 168 cm (*p* < 0.05).

Table 3 shows the percentiles distribution using the LMS method (p50, p90, p95, and p97) adjusted for height ranges of 10 cm for both sexes. The values for DBP and SBP increased as did the height ranges.

**Table 3:** Distribution of percentiles to assess DBP and SBP in children and adolescents adjusted for absolute height and sex

Height ranges (cm)	DBP (mmHg)							SBP (mmHg)						
	L	M	S	P50	P90	P95	P97	L	M	S	P50	P90	P95	P97
Males														
108-117.9	-0.64	60.3	0.18	60.3	77.6	84	88.7	-0.26	97	0.13	97	115.1	121.1	125.1
118 -127.9	-0.44	61.6	0.18	61.6	78.1	83.9	88	-0.09	99.1	0.13	99.1	117.4	123.2	127.2
128-237.9	-0.28	64	0.17	64	80.1	85.5	89.4	0	102.6	0.13	102.6	121.3	127.2	131.2
138-147.9	-0.16	66.3	0.17	66.3	82.3	87.6	91.3	-0.04	106.5	0.13	106.5	126.2	132.5	136.7
148-157.9	-0.09	68	0.16	68	83.8	89	92.6	-0.19	110.1	0.13	110.1	131.1	137.9	142.6
158-167.9	-0.01	69.6	0.16	69.6	85.6	90.8	94.3	-0.34	114.1	0.14	114.1	136.8	144.3	149.5
168-177.9	0.07	70.4	0.16	70.4	86.6	91.8	95.3	-0.27	116.1	0.14	116.1	139.9	147.8	153.2
178-187.9	0.11	70.4	0.17	70.4	86.8	92	95.5	-0.07	117.1	0.15	117.1	141.7	149.7	155.1
>188	0.14	70.5	0.17	70.5	87	92.2	95.8	0.14	118.4	0.15	118.4	143.7	151.7	157.1
Females														
98-107.9	-0.33	56.9	0.19	56.9	73	78.7	82.6	1.4	95.3	0.13	95.3	110.5	114.6	117.3
108-117.9	-0.31	59.1	0.17	59.1	74.4	79.7	83.4	1.34	97.7	0.13	97.7	113.2	117.4	120.2
118 -127.9	-0.27	60.5	0.16	60.5	75	79.8	83.2	1.18	99.7	0.12	99.7	115.3	119.7	122.5
128-237.9	-0.1	62.4	0.15	62.4	76.2	80.7	83.7	0.92	101.5	0.12	101.5	117.3	121.8	124.8
138-147.9	0.14	64.8	0.15	64.8	78.3	82.5	85.4	0.59	104.2	0.12	104.2	120.7	125.5	128.7
148-157.9	0.25	67.6	0.15	67.6	81.6	85.9	88.8	0.34	107.5	0.12	107.5	125.2	130.5	134
158-167.9	0.08	70.1	0.15	70.1	84.8	89.5	92.7	0.2	111.2	0.13	111.2	130.4	136.3	140.2
168-177.9	-0.15	72.3	0.15	72.3	87.8	92.8	96.3	0.02	115.9	0.13	115.9	137.1	143.8	148.3
>178	-0.39	74.4	0.15	74.4	90.6	96	99.8	-0.16	121.1	0.14	121.1	144.6	152.2	157.4

Legend: *M* medium, *L* Box-Cox transformation, *S* coefficient of variation, *P* percentile, *SBP* systolic blood pressure, *DBP* diastolic blood pressure

## Discussion

The results from this study have shown slight to moderate positive correlations between height with DBP and SBP categories of normotensive and hypertensive BP. These correlations were relatively similar in the height ranges determined by 5 and 10 cm. However, when comparing DBP and SBP by height range of 5 cm by sex, significant differences occurred in 4 height ranges (from 128 to 133 cm; 133 to 138 cm; 138 to 143 cm; and from 143 to 148 cm). In addition, significant differences were observed in the 10 cm range in only one of 138 to 148 cm in DBP and in SBP, height from 158 to 168 cm.

As a result, based on the findings obtained, the results from this research demonstrated that the BP differs very little in the height ranges of 10 cm. This appears to reflect a better suitability to evaluate DBP and SBP in children and adolescents in the Maule Region in relation to 5 cm.

In fact, in the 10 cm ranges, children and adolescents from various ages were grouped together to fit into particular height ranges. This allowed correcting for slow and/or rapid growth rates among children and adolescents. Thus, at whatever age, height may vary, resulting to a large extent in the presence of a wider range of BP [12] values. Therefore, the use of 10 cm height intervals to evaluate BP may be an advantage over chronological age since it is widely known that during the stages of childhood and adolescence that children and adolescents experience important changes in body size and maturation during physical growth [20]. Thus, height would explain variability substantially more than age [21].

In this sense, height is a practical and accurate measure that serves to evaluate a variety of populations and diverse ethnic groups during the growth stage, especially when it is used in conjunction with evaluating BP [12, 22, 23]. Furthermore, it appears to be immanent that efforts are being made to correct and create simpler and more practical techniques and tools to better identify hypertension in children [24] and adolescents and incorporate height routinely into medical examinations.

A number of studies have reported that height is a non-invasive alternative that serves to analyze changes and/or thresholds of BP related to chronological age [21]. Also, height is considered as a useful indicator for doctors. The use of this anthropometric variable may contribute to identifying children and adolescents with elevated BP and, consequently, offer a possible treatment [8].

In fact, based on previous findings, the researchers developed percentiles for DBP and SBP based on 10 cm height ranges for children and adolescents of the Maule Region. In effect, the United States Department of Health and Human Services [17] maintains that the reference values for BP thresholds for children and adolescents need to meet the requisites for six variables: DBP, SBP, age, gender, weight, and percentile for height.

The proposed percentiles for this study meet the requirements indicated above. This tool may serve as a simple and easy to use alternative for early detection of pre- and hypertension in children and adolescents. It may also be useful for professionals working in clinical and epidemiological contexts. In addition, it may have an important role in the prevention of cardiac [25] diseases during growth and development.

As a result, the cut-off points adopted for this research were those proposed in the fourth report of the US Department of Health and Human Services [17]: <p90 as normotensive; ≥p90 to p95 as pre-hypertensive; and ≥ p95 as hypertensive. These cut-off points determine limits and identify children and adolescents at greater risk of pediatric hypertension. The cut-off points also help identify individuals who need to have more examinations to control BP, including promoting preventative and healthy [2] lifestyles.

It is widely recognized that the reference standards for development for a specific population may not be applicable to other geographic regions. This is due to racial, ethnic, anthropometric, and cultural [26] differences.

In this sense, in a recent study carried out by other researchers [27], they determined that the students from the Maule Region reached adult height of 172.1 ± 6.9 cm for males and 159.8 ± 5.7 cm for females. In fact, these values correspond to a height range of the percentiles proposed here of 168–178 cm for males with a BP of 139.9/86.6 mmHg and for females, a height range of 158–168 cm with a corresponding BP of 130.4/84.8 mmHg. These values at 18 years old are close to the limits of 140/90 mmHg used for adults, coinciding with the values obtained in the present study.

Future studies need to evaluate not only height ranges, but also they need to explore ranges for weight, BMI, waist circumference, and among other anthropometric variables. Furthermore, it is necessary to develop longitudinal studies since height growth varies ostensibly, especially during the transition from childhood to adolescence.

As a result, despite the limitations highlighted here, this research has some strengths. For example, the probability selection of the sample makes it possible to generalize the results to other contexts with similar characteristics. In addition, we proposed the availability for professionals and researchers of an online electronic calculator system for evaluating BP by height ranges. The graphic reports provided by the calculations could significantly facilitate BP evaluations. The calculator may be obtained online with the following link: http://www.reidebihu.net/pad_pas_ch.php.

## Conclusions

In conclusion, the researchers identifies that height is a determinant for evaluating BP, and the height ranges for 10 cm are better suited for children and adolescents of the Maule Region. BP in the 10 cm height ranges differs very little from those of 5 cm. In light of these results, percentiles were proposed for evaluating DBP and SBP based on height ranges and sex. This information is useful for identifying children and adolescents with elevated BP and needs to be included in routine clinical examinations and in the educational system.

## Data Availability

The datasets supporting the conclusions of this research article are available by emailing the corresponding author.

## Abbreviations

BP: Blood pressure
DBP: Diastolic blood pressure
SBP: Systolic blood pressure
BMI: Body Mass Index
AAP: American Academy of Pediatrics
